# A systematic review of STEM teacher recruitment and retention interventions

**DOI:** 10.1186/s40594-025-00550-6

**Published:** 2025-07-01

**Authors:** Sophie Thompson-Lee, Beng Huat See, Robert Mark Klassen

**Affiliations:** 1 University of Oxford, Oxford, UK; 2 University of Birmingham, Birmingham, UK

**Keywords:** STEM, Science, Teaching, Retention, Recruitment, Systematic review

## Abstract

The worldwide problem of teacher recruitment and retention is particularly pronounced in STEM (Science, Technology, Engineering, and Mathematics) subjects, due in part to a lack of trainee teachers and to high rates of attrition. The teacher shortage has resulted in non-specialist teachers teaching STEM subjects and is likely to have a negative effect on the next generation of STEM students and STEM teachers. Numerous studies have outlined, and in some cases evaluated, recruitment and retention interventions, but to our knowledge a comprehensive review of interventions specifically aimed at recruiting STEM teachers has not yet been done. We reviewed 25 studies (9 recruitment, 11 retention, 5 both). Most interventions were financial (13/25), others included teacher education and alternative pathways. We evaluated study quality and the method each study used to assess intervention effectiveness. Financial incentives do not appear effective for recruitment despite being the most common incentive. Financial incentives seem to be more effective for retention; 3/9 higher quality studies found positive results. Findings for the other types of intervention were mixed and due to low design quality, not compelling. Our findings suggest that financial incentives might be effective for retention. Further research is needed to determine what interventions work for recruitment as the most common, financial incentives, do not appear effective. Studies investigating the efficacy of interventions need to be more rigorous with large sample sizes, comparison groups, and ideally randomised-control trials. There is also room for innovation as we did not find much evidence of novel intervention types.

## Introduction

In a world where we are expected to understand, and make informed decisions, about diverse topics such as climate change, global health, and alternative energy, alongside personal decisions about our medical treatment, choice of car, or use of artificial intelligence, science and technology education has never been more important. Against this context, there is a global shortage of teachers, especially in STEM (Science, Technology, Engineering, and Mathematics) subjects (See et al., 2022). Even in countries producing the most STEM graduates worldwide (Oliss et al., 2023), there are STEM teacher shortages. The top three countries for producing STEM graduates are China, India, and the United States of America (USA). In China, where there is high demand for STEM teachers and not enough people to fill the jobs, interest in teaching has also reduced since 2019 (Jacoutot, 2021). In India, 9% of STEM teacher posts were unfilled in 2022 (Education for All India, 2023) rising to 25% in some regions. In the USA, it is estimated that a third of states are experiencing a STEM teacher shortage (Jones II, 2023). Regarding interest in teaching as a career, only 40% of STEM undergraduates in the USA, and 30% in Australia and the United Kingdom of Great Britain and Northern Ireland (UK), would be willing to consider a teaching career (Elfers et al., 2008; Rice et al., 2023; Thompson-Lee et al., 2024). One consequence of these shortages is that non-specialist teachers are teaching STEM subjects to the next generation. A recent survey in the UK found that for both maths and physics, almost half of secondary senior leaders reported using non-specialists to teach some classes. (Worth & Faulkner-Ellis, 2022). Similar has been found in Australia (Weldon, 2016), Germany (Richter et al., 2019), Ireland (Ní Ríordáin & Hannigan, 2009), and the USA (Luft et al., 2013). The use of non-specialists may result in teachers who are underqualified (Hobbs, 2013; Ingersoll, 1999) and therefore ill-equipped to inspire the next generation of STEM graduates thus exacerbating the teacher shortage in the future (Rice et al., 2023). This systematic review aims to draw together attempts to improve recruitment and retention of STEM teachers by reviewing empirical studies that have designed and / or evaluated these interventions.

## Recruitment and retention interventions

Countries with teacher shortages have made targeted efforts to recruit more teacher trainees. For example, in the USA, trainees can receive loan forgiveness and cash awards for teaching in shortage subjects such as science (26 states offered this in 2016). In the UK, in 2023–2024, teacher trainees in chemistry, computing, mathematics, and physics were eligible for a bursary or scholarship if they had higher-than-average high school grades (DfE, 2025). In Australia, scholarships are available for all subject areas (Department of Education, 2025), and in a small initiative, the Teachers of STEM Initiative, Indigenous women are offered scholarships and training opportunities to train as STEM teachers in an effort to tackle a number of inequalities (Teachers of STEM Initiative (2025). In the USA, scholarships and bursaries are available to STEM teacher trainees through initiatives such as the Noyce scholarship and MASS (Math and Science Scholars Program).

Despite the prevalence of scholarships and bursaries, there is little evidence that they increase applications to teacher training (Allen and Sims (2017); Noyes, 2019; See et al., 2020). Furthermore, these approaches focus disproportionately on examination results (e.g. high school exams or university degree results) rather than on the skills, attributes, and values that make good teachers (Noyes, 2019) and additionally place heavy emphasis on initial recruitment rather than on retention.

Recruitment is only part of the problem; worldwide, teachers are leaving the profession at alarming rates. According to a recent United Nations Educational, Scientific and Cultural Organization (UNSECO) report, 58% of the need for teachers worldwide will be driven by attrition by 2030 (UNESCO, 2024). As pointed out by this report, this is a problem that effects all countries, including low- and high-income nations. For example, in the UK, a third of early career teachers have left teaching by their sixth year in the profession, and only 40.6% are still teaching after 25 years (DfE, 2023). Attrition has been found to be driven by job satisfaction, which in turn has been found to be predicted by many factors, for example, behavioural problems, SES, classroom discipline climate, school location, principal job satisfaction, school autonomy for instruction, participation among stakeholders, experience, teacher self-efficacy, teacher–student relationship, teacher cooperation, and effective professional development (as found by Wang et al., 2020 using data from the Teaching and Learning International Survey [TALIS] 2013).

Attrition is likely to be a greater issue for STEM teachers compared to teachers of other subjects because their skills and qualifications are highly transferable. An analysis of US data has found that teachers of mathematics and science, along with teachers of special education, English language, and foreign languages, are more likely to leave their school or the profession than teachers in other subjects (Carver-Thomas & Darling-Hammond, 2017). Furthermore, people in science careers typically out-earn those in non-science careers, such as teaching (Anlezark et al., 2008). Increasing recruitment to boost teacher numbers will only improve the situation to a limited extent; experienced teachers are needed to support early career teachers and research has found that experience is positively correlated with effectiveness (Podolsky et al., 2019). Teachers may most increase their effectiveness early in their careers but develop even further beyond the first decade in the classroom (Podolsky et al., 2019).

One reason for people leaving teaching is a lack of prestige and attractiveness of the profession, partly due to low salaries or irregular payments (UNESCO, 2024). In numerous countries around the world, teachers’ salaries are too low to allow for basic needs such as medical care (Katete & Nyangarika, 2020; Mpundu et al., 2023). Other reasons for people leaving teaching include workplace stress and burnout (Wang et al., 2019, 2022), fear of violence in the workplace (National Education Union [NEU], 2023), workload (NEU, 2023), lack of autonomy and intellectual challenge (Goldring et al., 2014; Rinke & Mawhinney, 2017; Smith & Ulvik, 2017), and quality of workplace relationships (Cui & Richardson, 2016).

Retention interventions have frequently been in the form of financial incentives. These incentives include salary increases for all teachers in a state or country, subject-specific (e.g. Science) or context-specific (e.g. high-needs) increases, and bonuses (upon employment or after time employed).

Salary increases aimed at increasing retention have ranged from being very ambitious (e.g. 25% per year over 4 years in Kazakhstan) to relatively more modest (e.g. a 6.5% one-time uplift in the UK). In some countries, financial incentives have been targeted at STEM teachers in particular. In the USA, teachers of maths and science have been offered 1800 American dollars (USD) bonuses for teaching, and remaining in, high-need schools (Clotfelter et al., 2008). In the UK, STEM teachers in disadvantaged schools receive £3,000 (3803 USD) tax-free payments per year over 3 years (DfE, 2022). In Australia, individual schools are offering as much as 10,000 Australian dollars (6298 USD) cash bonuses for physics and science teachers (Longbottom, 2022). Less well funded schools are unlikely to be able to compete with this and will likely continue to be short of teachers. Recruitment and retention interventions also include additional training opportunities and alternative routes into teaching.

Some interventions combine financial incentives with alternative pathways or training e.g. Teach for America, Noyce, and MASS. Teach for America combines alternative certification with salary and benefits when training in the form of “fellowships” (Teach for America, 2025).

The growing interest, and concern, about STEM teacher recruitment has led to numerous research studies investigating interventions for recruitment and retention. To date, there has not been a systematic review of this body of research to determine what interventions have been tested empirically and to assess the quality of the methodology and analyses used. A number of systematic reviews have been conducted on empirical research for teaching recruitment and retention broadly (see Guarino et al., 2006; See et al., 2020) while other reviews have focused on specific issues, such as hard-to-staff areas (Evans & Acosta, 2023; McPherson et al., 2024) and recruitment of principals (Lee & Mao, 2023). These reviews have found that financial incentives may be effective for retention, but less so for recruitment. Teacher education has also been found to be used as a retention intervention as part of continued professional development, but as found by See et al. (2020), evidence for these incentives’ efficacy is weak due to a lack of robust study design. In light of global pressures on the STEM teacher pipeline, there is a need to understand what works (and what does not work) in recruiting and retaining STEM teachers. The current review aims to build on previous work on general teacher recruitment and retention (e.g., See et al., 2020) by systematically reviewing studies evaluating STEM teacher recruitment and retention interventions.

## Intervention definitions and rationale

Based upon the literature outlined above and the studies included in this review, we group salary raises, bonuses, and payment to train as “financial incentives” in this paper. Salary raises and bonuses are conceptually similar as they increase pay in the pocket of teachers. In the papers in this review, salary raises and bonuses were employed as retention interventions. We acknowledge that salary increases may increase recruitment, but we did not find papers that empirically investigated this question. Payments to train, such as loan forgiveness or stipends, were used as recruitment interventions in the majority of the studies in this review.

As well as financial incentives, we found studies focused on the efficacy of alternative pathways or training. We used authors’ terminology to group papers as focusing on the efficacy of alternative pathways and /or training. In cases where the authors refer to the intervention as alternative certification, we defined this as an alternative pathway (e.g. Scott et al., 2006). Recruitment programmes, alternative certification programmes, and alternative pathways were all grouped as “alternative pathways” in our study, for example Abell et al. (2006) refers to a “programme” as an alternative route to certification.

Extra training (pre-service or CPD) and teaching practice/simulations were defined as “training” in this review, e.g. in Allen and Sims (2017) the National STEM Learning Network is referred to as CPD. The (Knowles Science Teaching Foundation) KSTF is an example of pre-service training as it offers extra training opportunities, such as community and collective inquiry into pedagogy and curriculum, to people already enrolled in teacher training (e.g. Galosy & Gillespie, 2013). Extra training (pre-service or CPD) and teaching practice/simulations are grouped together as “training” because they seek to increase self-efficacy through providing mastery experience (including vicarious and simulated) and as a result increase the likelihood of teachers entering and remaining in the profession (Watt & Richardson, 2007). Previous research has found that specifically for STEM teachers, CPD increases self-efficacy (see Zhou et al., 2023 for a meta-analysis of these studies).

## Research questions

The goal of our systematic review is to explore empirical literature that examines STEM teacher recruitment and retention. To achieve this goal, we raise three research questions:

- What are the most promising approaches in attracting STEM teachers into the profession?
- What are the most promising approaches in retaining STEM teachers in the profession?
- What is the current state of empirical research into STEM teacher recruitment and retention interventions?
- What are the implications for the design and implementation of future studies?

## Method

This review summarises the available research about interventions designed to enhance recruitment and retention of teachers for STEM subjects. To best inform policy makers about the most effective ways to respond to the lack of teachers, this review evaluates the quality of the evidence provided by each study.

The studies in the review were identified from a search of nine educational, psychological, and sociological electronic databases. These included:

- Web of Science
- Scopus
- ProQuest Dissertations & Theses Global
- EBSCOhost
- Sage
- JSTOR
- Cochrane Library
- Wiley
- Google and Google Scholar

**Fig. 1:**
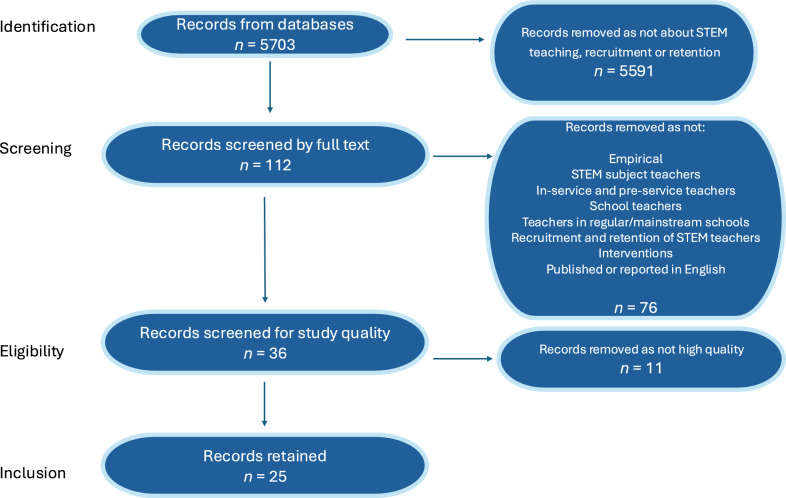
PRISMA diagram for systematic review

The search included the terms teacher recruitment, teacher retention, teacher attrition, and their synonyms, and science or STEM and their synonyms. Figure 1 presents the Preferred Reporting Items for Systematic Reviews and Meta-Analyses (PRISMA) diagram explaining the search process (identification, screening, eligibility, and inclusion). Each identified study was first screened to remove duplicates, and for relevance on the basis of title and abstract. Only studies that related specifically to recruitment and retention for STEM/science teachers were included. This process removed 5591 studies, leaving 112 which were read in full. We screened the studies applying the inclusion and exclusion criteria below. We did not limit for publication year or geographical region of the research. Studies were included if they were:

- Empirical
- STEM subject teachers
- In-service and pre-service teachers
- School teachers
- Teachers in regular/mainstream schools
- Recruitment and retention of STEM teachers
- Interventions, activities, policies or strategies aimed at attracting people into teaching or about retaining teachers in teaching.
- Published or reported in English.

After screening for relevance, 36 studies remained. We then assessed the strength of evidence provided by each study using ‘the sieve’ process (Gorard, 2018)—a tool for judging the trustworthiness of research findings. If a review, such as this one, aims to inform policy makers about recruitment and retention interventions and their efficacy, then ensuring the evidence is trustworthy is critical. Basing education policy on incorrect, misleading, or incomplete evidence may be the reason that many interventions are not effective (See et al., 2020).

Following Gorard (2018) and See et al. (2020), the studies were assessed using five criteria: the research design (e.g. whether the design was appropriate for a causal claim, such as an RCT with random assignment of cases and whether there was a comparison group), scale of the study (smallest cell size), level of attrition, validity of outcome measurement (e.g. administrative data versus teacher self-report), and other threats to validity (conflicts of interest). Each study was given a score between 1* (the minimum standard to be given any weight, including some kind of comparison) and 4* (the most robust that could be expected in reality). Four-star studies are the strongest, meaning that the evidence is most reliable. For more detailed information on using ‘the sieve’ in a systematic review, see See et al. (2020).

We reviewed the 36 retrieved articles for design quality (data available on the OSF repository. See Declarations for URL). Eleven studies received a score of 0* and were not included in the final review. These studies were assessed as providing weak evidence due to using self-report of intention to stay in teaching, very small sample sizes (e.g. *n* = 11), and/or no comparison group.

Nine studies were rated 1*. These were rated 1* rather than 0* because some of them used a comparison group but only collected self-reports, or the sample size was large, but no comparison group was used, or data were based on self-reports. We included the 1* studies in the final review because we wanted to assess the quality of as much empirical research as possible, and nine studies made up a substantial proportion of our sample.

Studies rated at least 2* had large sample sizes, comparison groups, and/or used pre-specified measures of career intention. None of the studies we found were rated 4* (i.e. none were randomised-control trials). Our review of study quality resulted in 25 studies remaining for further analysis (see Appendix for study details).

## Synthesis

The included studies were grouped according to whether they were about recruitment or retention. Where studies were about both, they were discussed under recruitment and retention. We then classified them broadly under types of interventions. These included financial interventions, alternative pathways to certification, and training support. Financial incentives included any interventions involving the use of monetary incentives (e.g. scholarships, bursaries and bonuses). Alternative pathways to teacher certification referred to non-traditional routes that allow individuals to be trained and certified without following the conventional university-based education programmes. For example, expedited certification where students complete training in a shorter duration (e.g. emergency or temporary certification) or where individuals (such as teaching assistants or paraprofessionals) already working in the school obtain certification while working on the job. In the case of STEM teaching, alternative pathways were often targeted at science majors prior to completion of college or university. This could be in the form of teacher training alongside a science major or placements or summer programmes. Teacher candidates can be recruited at all career levels, for example, direct from high school, transfer from community college, change of major, and career changers. Examples of alternative pathways include Teach for America, Teach First, school-based teacher training (e.g. School Centred Initial Teacher Training), residence-based programmes where teacher candidates co-teach with experienced teachers while earning certification. Training support includes mentoring, induction and professional development.

Some training programmes may involve more than one type of intervention. For example, the Robert Noyce progamme provides scholarships, stipends and professional development. We categorised studies based on the primary focus of the study and the research questions.

## Results

Nine of the 25 studies in our sample assessed interventions designed to recruit STEM teachers. Eleven of the 25 investigated only retention interventions. Five investigated both recruitment and retention interventions. In the following analyses these studies are counted twice, so there are 14 outcomes for recruitment and 16 for retention. For both types of intervention (recruitment and retention), the majority were financial incentives (recruitment = 8/14, retention = 10/16). Most of the studies in our sample were from the USA, and four were from the UK. The dates of the studies ranged from 2000–2024.

Six of the studies investigated interventions specifically for high-needs schools. We include these in our review because they aimed to recruit or retain STEM teachers. Only one study that focused on retention analysed whether teachers left the profession altogether or moved into a different setting. Previous research is mixed as to whether teachers are more likely to leave high-needs settings than other settings (Arthur & Bradley, 2023). The studies in our sample do not consistently measure retention, for instance, one study defines retention as staying in the same school whereas another in the same district. Whether it be in a high needs setting or another setting in most cases we were unable to say where teachers went when they left. We acknowledge that caution must be taken when comparing interventions aiming to recruit or retain teachers in high-need settings with those in other settings. In our sample, this affected only a small number of studies: one focusing on recruitment, two on both recruitment and retention, and three on retention. Three of these investigated financial incentives, two training, and one alternative pathway.

## Recruitment interventions

Two of the studies focusing on recruitment interventions were in the UK, the others were from the USA (12). The majority of studies examined financial incentives to recruit teachers, including scholarships (8/9), and a subject-specific bonus scheme (1/8). Three studies focused on alternative pathways to teaching and three used pre-service training as an intervention. Two studies were counted twice (Demir et al., 2019; Scott et al., 2006) as they evaluated two types of intervention (financial incentives and an alternative pathway).

Pre-service training interventions included realistic job previews and teaching simulation, and master’s level pre-service training. Two studies used interventions which were categorised as a teaching simulation (Vignettes, Thompson-Lee et al., 2024; RJPs, Klassen et al., 2023). One study assessed the effect of mentoring on recruitment as part of extra support given during teacher training. Since these three studies provided realistic and authentic insights into teaching, beyond typical teacher training provision, we grouped them together as “training”. Both RJPs and mentoring theoretically are intended to increase teacher self-efficacy through providing vicarious experience and positive feedback (from simulations or teacher anecdote, respectively) (Bandura, 1997). Although the number of studies using these types of intervention is too small to make confident conclusions it could be argued that they are designed to increase recruitment through similar theoretical means.

### Use of financial incentives

Financial incentives remain the most often used, or most researched, incentives to attract STEM teachers. However, there was mixed evidence about their effectiveness, with none of the 2* + studies reporting uniformly positive outcomes, and three of the studies finding negative or neutral outcomes. The evidence rating for these studies is summarised in Table 1.

**Table 1:** Evidence rating of studies on recruitment using financial incentives (*n* = 9)

Strength of evidence of study	Positive outcome (*n* = 4)	Mixed/inconclusive outcome (*n* = 2)	Negative or neutral outcome (*n* = 3)
3* (*n* = 1)			Bueno and Sass (2018)
2* (*n* = 2)		Marder et al. (2022)	Templeton et al. (2023)
1* (*n* = 4)	Demir et al. (2019) Morrell and Salomone (2017) Scott et al. (2006) Urdegar (2019)	Zahner et al. (2019)	Whitfield et al. (2021)

The study with the highest rating (3*) (Bueno and Sass (2018)) found that financial incentives had no effect on recruitment. This study investigated the effect of a bonus system to differentiate teacher pay based on subject area for maths and science teachers. Using a difference-in-difference approach, the authors found no evidence that the bonus scheme increased the likelihood that education majors would become secondary maths or science teachers after completing their studies. Two other studies also found no effects of financial incentives on recruitment. These were not of as high quality and were rated 1* (Whitfield et al., 2021) and 2* (Templeton et al., 2023).

Templeton et al. (2023) and Whitfield et al. (2021) investigated the effectiveness of the Robert Noyce Teacher Scholarship Program (RNTSP) in increasing the placement and retention of STEM teachers. The RNTSP (also known as Noyce), investigated in six of the studies in our sample, is a US based programme that offers 10,000 USD stipends for scholars pursuing graduate-level, initial teacher certification in any STEM field at the secondary level.

Templeton et al. (2023) used a difference-in-difference approach to compare Noyce participants with non-Noyce participants who were on teacher education programmes and had taught at least one middle school or high school STEM course for one school year. This study particularly focused on high-needs schools in Texas, which were defined as serving at least 75% low-income students. Noyce teachers were found to be no more likely to teach STEM in the highest need areas (34%) compared to non-Noyce teachers (38%). Whitfield et al. (2021) collected longitudinal data from 29 Noyce Scholars to investigate the influences the scholarship had on recruitment of STEM teachers in high-need settings. Three questionnaires were administered over 3 years alongside interviews. The programme was found to have no impact on decision to teach. The majority of the recipients (74%) did not think the programme had influenced their decision to become a teacher, to complete the teacher certification programme (70%), or to commit to a teaching job (67%).

The four studies in our sample that found evidence of positive outcomes of financial incentives were studies rated 1* (a priori) for research quality (Demir et al., 2019; Morrell & Salomone, 2017; Scott et al., 2006; Urdegar, 2019). Morrell and Salomone, (2017) focused on the Noyce scholarship programme and used questionnaires and focus group interviews to survey interns before and after their teaching experience and a focus group interview at the end of their internship. The programme appeared to have some influence on STEM undergraduates' decisions to be teachers. All scholars who completed the program were teaching in high-need schools, but there were only 16 participants in the sample. There was no comparison group and the role of the programme in the participants’ decision to teach was determined using self-report, therefore, it is not possible to be sure that the programme was the determining factor in the participants’ decision to become teachers.

Scott et al. (2006) similarly found that numbers of trainees recruited into the first year of training increased, but only 59% continued into year two of training. Again, no comparison group was used so this effect may not have been solely due to the scholarship programme under investigation (MASS). Urdegar (2019) also did not use a comparison group and relied only on descriptive statistics as to whether teacher trainees were placed in STEM subject areas. The study found that of 170 trainees, 25 were assigned to teach maths, 18 science and 40 to teach a combination of subjects including maths and this was considered to be a positive outcome for recruitment into STEM teaching. The final study to find a positive effect used self-reported commitment to teach as their measure of intervention efficacy (Demir et al., 2019). Fifty-seven/63 trainees reported an intention to teach after their training, but this was not confirmed by actual numbers of trainees who entered teaching posts.

### Alternative pathways

The next most popular intervention that was evaluated in the studies we reviewed is alternative routes into teaching. For example, in England, there are multiple ways people can train for teaching, e.g. School Direct (a programme for career changers), postgraduate apprenticeship schemes, returning teachers schemes, and the Researchers in School programme for those with a doctorate or completing a doctorate in maths and physics, with all of these pathways aimed at providing training on the job. However, to our knowledge, none of these programmes have been evaluated in peer-reviewed reports, at least as found in our search of peer-reviewed research publications. Alternative programmes are designed to enable those who have not met the entry requirements into teaching via the traditional university routes and they allow direct entry to teaching therefore providing an opportunity to simultaneously earn and study. In the US, the Pathways to Teaching Careers Program (PTTCP) funded by the Wallace Foundation is a well-known example, but again, peer-reviewed research documenting its effectiveness is scarce.

Our review found no clear evidence that alternative pathways to training were any more effective than traditional routes (see Table 2). The strength of evidence of the studies is rated as generally weak, and although these studies did indicate an increase in recruitment, it was unclear if this was due to the financial penalty incurred or the heightened recruitment efforts.

**Table 2:** Evidence rating of studies on recruitment using alternative pathways (*n* = 4)

Strength of evidence of study	Positive outcome (*n* = 3)	Mixed/inconclusive outcome (*n* = 1)	Negative or neutral outcome (*n* = 0)
3*			
2* (*n* = 1)		Bowe et al., (2011)	
1* (*n* = 3)	Abell et al. (2006) Demir et al. (2019) Scott et al. (2006)		

The highest rated study to evaluate an alternative pathway was rated 2* and found inconclusive results (Bowe et al., 2011). This study focused on Noyce scholars in traditional and alternative programmes and assessed their commitment to teach. Descriptive statistics suggested that participants in the alternative pathway were more likely to indicate that they would have taught in a high-need school even without the Noyce funding than those in a traditional programme (41% compared to 36%), but this difference was not confirmed by inferential statistical testing. This study controlled for financial incentives as all participants received Noyce funding and included a comparison group, therefore its findings are more robust than the other studies focused on alternative pathways in our sample, but the lack of statistical significance suggests that the alternative pathway did not have a strong effect on student decision-making about teaching as a career.

The other three studies were of lower quality (1*) and found positive effects of using alternative pathways. Abell et al. (2006) aimed to evaluate the SMAR2T program (SMAR2T: Science and Mathematics Academy for the Recruitment and Retention of Teachers). The SMAR2T program was funded by the National Science Foundation in the US and aims to offer alternative pathways to teaching for postbaccalaureate students. The programme consists of two options to achieve teaching accreditation; one in which participants are full-time students who completed the program in 15 months and the other in which participants are full-time teachers who completed the program in 24 months. This study found that enrolment on the course increased following heightened recruitment efforts. It is unclear whether the increase in recruitment is due to the alternative pathway itself or due to the recruitment methods used (including pointing out scholarship opportunities separate to the programme, e.g. Noyce).

The second study to find positive results also presents an unclear picture. The Teach Science and Math programme (TSM), evaluated by Demir et al. (2019), was a federally funded science and maths teacher education recruitment programme involving an alternative certification pathway and a scholarship with golden handcuffs. The majority of the participants (57/63) committed to teaching after the programme, or they would have to repay costs. It was unclear to what extent the alternative pathway element increased this commitment rather than the financial penalty.

Finally, Scott et al. (2006) found positive effects of a teacher recruitment programme on recruitment (MASS). Participants in the programme were also recipients of a scholarship. It was unclear whether the financial incentive, or the training, was the main cause of recruitment and retention on the course.

### Training

Two studies in our sample used training as a recruitment intervention. The training was designed to expose potential future teachers to teaching simulations to enhance their knowledge of teaching in order to increase interest in teaching and their self-efficacy for teaching. One study showed positive effects while the effect of the other study was inconclusive (Table 3).

**Table 3:** Evidence rating of studies on recruitment using training (*n* = 2)

Strength of evidence of study	Positive outcome (*n* = 1)	Mixed/inconclusive outcome (*n* = 1)	Negative or neutral outcome (*n* = 0)
3*			
2* (*n* = 2)	Thompson-Lee et al. (2024)	Klassen et al. (2023)	
1*			

Using a novel approach adopted from organisational psychology, Klassen et al. (2023) designed, implemented, and tested a STEM teacher attraction intervention based on person–environment (specifically person–vocation [PV]) fit theory. This study designed a realistic job preview (RJP) intervention and measured its effects on 111 university students’ interest in teaching as a career, self-efficacy for teaching, and the perceived match between personal attributes and those attributes required to be a teacher. A significant association between RJP scores and interest in a teaching career and person–vocation fit was found, even when controlling for prior career intentions. This study did not follow the undergraduates’ actual career paths so the effect on recruitment cannot be assessed. Therefore, the outcome was inconclusive regarding actual decisions to enter teacher training.

Building on the concept of RJPs, Thompson-Lee et al. (2024) designed a vignette intervention to expose 423 undergraduates in the UK to realistic teaching scenarios that evoked utility values and PV fit. The intervention was found to increase interest in teaching significantly more than a control group. As in Klassen et al. (2023), this study did not track the participants longitudinally and so actual recruitment was not measured.

## Retention interventions

Sixteen studies investigated retention interventions. The retention intervention studies were more robust than the recruitment intervention studies in terms of study quality. Seven of 16 were rated 3* compared to one of the 12 recruitment focused studies. The retention interventions investigated included financial incentives (*n* = 10), alternative pathways (*n* = 5), and preservice and in-service training (*n* = 4). One study focused on a financial incentive paired with alternative pathway and two on a financial incentive paired with training (these are counted twice; once in each category).

The majority of studies did not differentiate between different types of attrition, e.g. movers and leavers (15/16). Additionally, it was often not clear whether retention referred to retention within schools or within the profession. Six studies discussed different types of attrition, but did not differentiate in their analysis. For this reason, we define attrition as anyone who has left, regardless of whether they have left the school, moved to another district or state, or left the profession.

### Financial incentives

Four studies that investigated financial incentives effect on retention were rated 3* (see Table 4). Three of these reported positive effects (Bueno and Sass (2018); Clotfelter et al., 2008; Feng & Sass, 2018), while one showed no consistent effect on retention (Glazerman & Seifullah, 2012).

**Table 4:** Evidence rating of studies on retention using financial incentives (*n* = 9)

Strength of evidence of study	Positive outcome (*n* = 4)	Mixed/inconclusive outcome (*n* = 1)	Negative or neutral outcome (*n* = 4)
3* (*n* = 4)	Bueno and Sass (2018) Clotfelter et al. (2008) Feng and Sass (2018)		Glazerman and Seifullah (2012)
2* (*n* = 3)	Sims and Benhenda (2022)		Marder et al. (2022); Templeton et al. (2023)
1* (*n* = 2)		Zahner et al. (2019)	Urdegar (2019)

Five studies focused on the bonus incentive schemes. The other four focused on scholarships. Scholarships included the Noyce scholarship and Teach for America. The majority (3/4) evaluated the Noyce scholarship. Two reported no effect (Marder et al., 2022; Templeton et al., 2023) and one suggest mixed effects (Zahner et al., 2019). Teach for America was found to have no effect on retention (Urdegar, 2019).

Bonus schemes appear to be more promising. Bonus schemes include one-off payments for remaining in a school for a number of years or payments for teaching particular subjects (e.g. maths or science) or in particular contexts (e.g. high-need schools). Four reported positive effects on retaining teachers, with three rated 3*, and one reported inconsistent results across years; positive effects were found for the first year, but not subsequent years (Glazerman & Seifullah, 2012), suggesting promising evidence of bonus incentives in retaining teachers.

Clotfelter et al. (2008) found that an annual bonus of 1800 USD for maths, science, and special education teachers working in public secondary schools with either high-poverty rates or low test-scores resulted in a one-sixth reduction in turnover rates.

Using a difference-in-difference approach to compare teachers who were eligible for a bonus with those who were not, Bueno and Sass (2018) found that the bonus resulted in a substantial reduction in attrition rates for secondary math and science teachers. This was a statewide program involving 31,171 teachers.

Another 3* study reported that the retention bonus offered to high school teachers in designated subject areas decreased teacher attrition by as much as 25% (Feng & Sass, 2018). Sims and Benhenda (2022), rated 2*, also reported a 25% reduction in attrition of a bonus scheme offered to STEM teachers.

Glazerman and Seifullah evaluated the impact of the 4-year Chicago TAP (Teacher Advancement Program), which provides mentoring, leadership opportunities, and financial incentives to schools and teachers on student academic achievement and teacher retention. Based on teachers’ performance on skills and responsibility, teachers receive an average bonus of 1000 USD in the first few years of implementation rising to 1400 USD in later years. The study utilised propensity score matching to compare retention of TAP schools and non-TAP schools. No consistent effect was found on teacher retention at either the school or district level. Positive effects on retention were noted for teachers in the first year of implementation. Teachers in Chicago TAP schools were 20% more likely than teachers in the comparison schools to be in those same schools 3 years later, but not in subsequent years. Although there was evidence of a positive effect of TAP on the retention of less experienced teachers, the pattern was not consistent across districts. This intervention included a training element too and so it is not possible to attribute the effect to only financial incentives.

The nine studies evaluating financial incentives provide a mixed picture, the strongest quality evidence suggests that financial incentives can work for retention, specifically in the form of bonuses. However, the majority of the studies (five/nine) suggest otherwise, particularly for the effects of scholarships.

### Alternative pathways

There are only five studies that evaluated the impact of alternative pathways on retention of STEM teachers (Table 5). In general, alternative pathways to teaching did not have a long-term effect on retention of STEM subject teachers.

**Table 5:** Evidence rating of studies on retention using alternative pathways (*n* = 5)

Strength of evidence of study	Positive outcome (*n* = 0)	Mixed/inconclusive outcome (*n* = 2)	Negative or neutral outcome (*n* = 3)
3* (*n* = 3)		Brantlinger and Grant (2022a); Boyd et al. (2012)	Brantlinger and Grant (2022b)
2* (*n* = 1)			Cooley (2009)
1* (*n* = 1)			Urdegar (2019)

The highest rated studies (Boyd et al., 2012; 3*) found that recruiting mathematics teachers from non-mathematics backgrounds using a math immersion program had mixed effects on retention. Initially, teachers from the maths immersion program had lower attrition rates compared to teachers from other pathways, but by year four, their rates had surpassed other teachers. So, positive effects were short-lived. Brantlinger and Grant (2022a) investigated the effect of the New York City Teaching Fellows (NYCTF) programme on maths teacher retention. This included master’s level instruction, coursework, a minimum of 40 h of practice teaching in a summer school classroom, and 40 h of NYCTF-delivered training. The findings were positive for Black/Latinx students but not for other demographics. Another 3* study, also exploring the effect of the NYCTF programme, revealed that the programme failed to improve mathematics teacher staffing and retention issues. However, it found that some aspects of the programme, such as such as, first-year induction, regular meetings with a mentor, and high-quality field work advisories, helped reduce attrition. These two studies investigated the efficacy of the same programme and so their results have limited generalisability despite the quality of the studies.

Our review also found no evidence that the Teach for America pathway to teaching had any long-term effect on the retention of STEM teachers. Urdegar (2019) found that most Teach for America (TFA) teachers had left their school by the 5-year mark. This study recorded attrition in the Miami area only, so it is possible these teachers did not leave teaching entirely but continued elsewhere. The lack of data on out of state teaching makes the attrition rate in this study unreliable, but does show that TFA does not work in keeping teachers in specific geographical areas once the term of their contract ends. Finally, Cooley (2009) found that maths teaching fellows left teaching at a higher rate than those entering through traditional pathways suggesting a negative effect of an alternative pathway.

The authors of many of these studies describe the programmes they investigated as alternative routes or paths to teaching, but these studies could arguably be investigating the effects of training since the programmes in question involve extra training and instruction. In this review, we evaluate studies on the basis of the terminology used by the authors but acknowledge that there is overlap in a number of studies with regard to their focus, i.e. alternative pathway or training.

### Training

There is no strong evidence that additional training of teachers via continuing professional development (CPD), or support of new teachers through induction and mentoring improves retention. The highest quality study (3*) in our review found that the Chicago TAP programme, which provides mentoring and leadership opportunities alongside financial incentives (outlined above), had no clear effect on retention (Glazerman & Seifullah, 2012) (Table 6).

**Table 6:** Evidence rating of studies on retention using training (*n* = 4)

Strength of evidence of study	Positive outcome (*n* = 2)	Mixed/inconclusive outcome (*n* = 0)	Negative or neutral outcome (*n* = 2)
3* (*n* = 1)			Glazerman and Seifullah (2012)
2* (*n* = 3)	Allen and Sims (2017) Galosy and Gillespie (2013)		Kirchhoff and Lawrenz (2011)
1*			

Positive effects were found by Galosy and Gillespie (2013). They found that the KSTF, which offers pre- and in-service training to new teachers, had higher retention rates compared to the US average, which ranges from 83–86% after the first year of teaching to 54–55% after the fifth year of teaching. KSTF teacher retention rates ranged from 96.9% following the first year of teaching to 88.8% following the fifth year. A caveat to these high retention rates is that this study’s evidence is not as robust as the previous 3* study (i.e. Glazerman & Seifullah, 2012) because it did not recruit a comparison group for its survey methodology and instead compared to national data.

In one of the few studies in our sample outside of the US, Allen and Sims (2017) assessed retention of UK-based science and non-science teachers to evaluate the effect of the National STEM Learning Network (NSLN) subject-specific CPD courses. Using national data, this study found that retention for teachers who had taken NSLN courses was 48% higher one year after first participating and 27% higher 2 years after first participating than teachers who did not take the courses.

Since those who plan to stay in teaching are more likely to participate in professional development courses as they have much to gain from such training, comparing the retention rates of participants with non-participants may give a misleading picture. To mitigate such unobserved differences, Allen & Sims used propensity score matching, matching participants with non-participants by known characteristics. However, propensity score matching only compared observed differences. To control for unobserved differences, comparisons were made between those who participated in 2010 with those who participated later. The individuals are therefore more likely to be similar in terms of motivation, career plans, and supportiveness of management in the department. Further analyses were also made comparing the science departments before and after the treatment. This study was not rated 3* because 11% of the cases were missing and could not be matched, but the large sample size and the mitigation for unobserved difference make the findings promising.

## Discussion

### What are the most promising approaches in attracting STEM teachers into the profession?

Most of the interventions designed to attract teachers in our review used financial incentives. This is an expected finding since previous reviews of teacher recruitment have found this to be the case (See et al., 2020). Most of these studies (four/nine), found mixed, negative, or no effect. Those that did find positive effects were rated 1* for research quality, the lowest score included in our review. Contrary to previous findings (See et al., 2020), the higher-rated studies in our sample suggest that financial incentives are not very effective for recruiting STEM teachers. This could be because of the specific career motivations of STEM graduates.

STEM graduates are more likely than graduates of other subjects to consider teaching as a fall-back career (Watt et al., 2017). They are also likely to have a wide range of well-paid jobs available to them, more so than for other graduates (Anlezark et al., 2008; Cambridge Industrial Innovation Policy, 2022; Kunz et al., 2020; Noonan, 2017). In Thompson-Lee et al. (2025), significant motivational differences were found between STEM and non-STEM high school students. STEM students were more motivated by personal utility values (salary, prestige, promotion) than non-STEM students. STEM students were also significantly less interested in teaching compared to non-STEM students.

Furthermore, in most countries, and particularly in the USA and UK where the studies in our sample were based, there is still an over-representation of men in STEM degrees (e.g. 73% of STEM graduates in the UK identified as men in 2021/2022, HESA [Higher Education Statistics Agency]), with implications for recruitment and retention. Research has found men to consider teaching a fall-back career more so than women (Gratacós et al., 2017; Watt et al., 2012; Wyatt-Smith et al., 2017). Women have been found to be more motivated by social utility factors than men (e.g. contributing to society, working with children), and for people who do enter teaching, social utility is often a driving factor as opposed to personal utility (e.g. financial gain) (see FIT-choice research, e.g. Watt & Richardson, 2008; See et al., 2022).

The majority of the research cited here was carried out with people who have already chosen to enter teacher training. Research investigating career motivations prior to entering higher education has found that female students are more likely to be interested in teaching than men (Gore et al., 2016; Klassen et al., 2025; Lai et al., 2005; Van Rooij et al., 2020) and that female high school students are more interested in teaching than other STEM or non-STEM careers (Fuchs et al., 2022). It should be noted, concerningly, that in a recent study in the UK mean interest in teaching was low for *both* men and women (Klassen et al., 2025).

Therefore, financial incentives, which provide personal utility, might not be compelling enough a reason to consider entering teaching, particularly for STEM students and men, and STEM graduates can likely receive higher financial payback from other STEM jobs meaning that financial incentives are unlikely to be sufficient to persuade people motivated more by personal utility values. See et al. (2020) found that financial incentives work only if they are large enough to compensate for the salary boost that these STEM graduates would receive from comparable professions.

The other intervention types that were evaluated, alternative pathways and additional training, did not offer compelling evidence of their efficacy. The highest quality studies (Klassen et al., 2023; Thompson-Lee et al., 2024), demonstrated that job previews are promising for increasing interest in STEM teaching. A well-established body of research into RJPs, which depict realistic snapshots of the pros and cons of a teacher’s life, has shown that RJPs delivered before training or employment can promote early integration into a new field, leading to better retention rates and job success (Baur et al., 2014). However, the long-term effectiveness of RJPs has not been tested with those in, or considering, teaching roles. The two studies (Klassen et al., 2023; Thompson-Lee et al., 2024) using previews of teaching conducted with prospective teachers did not follow STEM undergraduates into initial teacher training so the effect of teaching simulation on actual recruitment and retention cannot be evaluated. Studies like Klassen et al. (2023) and Thompson-Lee et al. (2024) that additionally follow participants to see if they enter teacher training, and then stay in teaching, would provide more robust evidence for the efficacy of this type of intervention. It is worth noting that, compared to financial incentives, RJPs are cheap and scalable, and have been found to be effective in other fields (Baur et al., 2014). Our review found that for the financial incentives investigated they did not appear to work for STEM graduates specifically, and furthermore, are expensive at scale. Therefore, novel approaches to attraction, like RJPs are likely to be worth pursuing.

### What are the most promising approaches in retaining STEM teachers in the profession?

We found a greater number of high-quality studies investigating retention than recruitment; seven retention studies were rated 3* compared to only one 3* recruitment intervention study. This means that the evidence provided by the high-quality retention studies is reasonably reliable and informative for policy makers and researchers. Financial incentives seem to be a promising intervention for retention and were the most frequently investigated. The majority of the studies that found positive effects were rated 3* or 2*. The most promising financial incentives were bonuses for science and maths teachers. However, the side effects of these bonuses on other subject teachers within the same system have not been investigated. These selective bonuses may cause resentment and demoralisation leading to higher attrition among other teachers. This is a concern in nations where this approach is used or is being considered. An example is the UK’s 2022 initiative to give STEM teachers in disadvantaged schools £3,000 (3803 USD) tax-free payments per year over 3 years (DfE, 2022). No evaluation of the potential side effects has been published. Furthermore, the longevity of the effects of bonuses is not explored by the majority of the studies in this review. Positive effects on retention may reduce over time resulting in the need for future bonuses.

There was not enough quality evidence to evaluate the other types of retention approaches (e.g. alternative pathways and training). The studies that investigated training found little or no effect on retention. Alternative pathways were also found to have little or no effect on retention. In some cases, alternative pathways and training were accompanied by financial incentives and so any effect could be due to one or a combination of interventions.

The studies in this review did not report what career teachers leaving the profession went on to pursue. Understanding where teachers go when they leave teaching is particularly relevant for STEM teachers as they are likely to have more career options available to them than teachers from other subject areas. In many studies, attrition could mean leaving the state the teachers were trained in, or working in, rather than leaving teaching entirely. Previous research has found that many teachers leaving teaching stay in education (e.g. Amitai & Houtte, 2022; Elsayed & Roch, 2023; Larsen et al., 2025), for example as teacher educators, this might be considered as a positive move compared to teachers leaving education entirely. More clarity on the career paths leavers take is needed to be sure of the effects of retention interventions. In order to understand career trajectories outside of teaching, longitudinal studies are needed that follow leavers as well as those staying in teaching.

### What is the current state of empirical research into STEM teacher recruitment and retention interventions?

There has been more research on retention interventions of STEM teachers than on recruitment interventions. This imbalance may be because governments and policy makers have focused more on retention, or it may be that researchers have been more interested in retention than recruitment. Most of the studies in our sample were rated 1* and 2* for research quality. None of the studies in our sample were rated 4*. A 4* study would be a randomised-control trial, have a large number of cases with low attrition, use a standardised and pre-specified method of data collection, and have little evidence of any other threats to reliability (Gorard, 2018).

Some of the lower rated studies are somewhat unreliable because they rely on self-report for retention intentions. In other words, they judge the efficacy of an intervention based on whether teachers say they will stay in the job. We do not know if these teachers actually remain in teaching. In the studies in the USA (the vast majority of our sample) teachers leaving a school or state often constitutes leaving teaching. In reality, teachers may have moved state/district and continued teaching elsewhere. Although some studies mention this issue, we found only one study that differentiated types of attrition in the analysis (Allen & Sims, 2017). There needs to be clearer distinction between attrition (leaving teaching) and turnover (leaving school/district/state but remaining in teaching) as seen in research into quitting intentions (e.g. Klassen et al., 2023). The latter would not constitute a wastage, but simply a re-distribution of teachers, albeit it potentially resulting in greater issues for certain types of schools, such as, high-need schools.

A number of studies investigated programmes such as TFA and the Noyce scholarship. These programmes sometimes combine financial incentives with other interventions, such as training. The effects of the different types of intervention in these studies is difficult to unpick. Studies controlling for other intervention types or comparing between groups with multiple or single interventions would give more robust insights into their effects.

We found very little evidence of innovation in terms of intervention design. Very similar scholarship and financial incentives programmes were used in the majority of studies. The two studies which showed the greatest innovation were those using realistic job previews/vignettes (Klassen et al., 2023; Thompson-Lee et al., 2024). However, these focused on increasing interest into STEM teaching and did not follow participants to find out if they actually entered teaching training.

## Conclusions and limitations

For retention, financial incentives appeared to be the most promising intervention type with the caveat of these studies being mostly carried out in individual US states and with none having very robust study design (e.g. no randomised-control trials). For recruitment, due to a lack of high-quality studies, it is difficult to select one type of intervention as being most likely to be successful. It must be noted that, based on the evidence we found, financial incentives do not appear to work well for STEM teachers, contrary to findings from a review into incentives for attracting teachers into hard-to-staff areas (See et al., 2020).

More research is needed that can determine causality in order to understand which interventions work particularly when used in combination with other interventions. Randomised control trials would allow for different intervention effects to be unpicked. For example, by comparing a group receiving a financial intervention with a group receiving another type of intervention and a control group receiving no intervention, it could be possible to explore the effects of each intervention, at least in the short-term. The long-term effects of interventions have only been explored in a few cases. Longitudinal designs are needed to understand career trajectories particularly in cases where teachers have been recorded as leaving teaching, but in fact may have continued teaching in a different geographical area. For interventions to be worth the costs, both financial and time, their efficacy needs to be shown longitudinally. Following teachers who have received training or financial incentives over longer periods of time, 10 years or more, is needed to really assess those incentives’ efficacy.

## Limitations

We endeavoured to make this review comprehensive but recognise that in any review of this scale some studies may have been missed, and new and more robust studies may be conducted in the future. We also acknowledge that the studies included in this review were those only published in English which may have limited the scope of our sample and results in a focus on Western, Educated, Industrialized, Rich and Democratic (WEIRD) nations. In our study, this means an over-representation of studies from the USA. More research is needed to evaluate interventions from more varied contexts.

Furthermore, we excluded studies based on their research design. This was done to ensure that only robust findings were synthesised in this review, in order to increase its usefulness for policy makers in particular. It must be acknowledged that, despite using the “Sieve” (Gorard, 2018) there is an element of subjectivity to rating research design. Whilst we can be confident that there were no studies included in our review that met the 4* criteria (a randomised-control trial, with a large number of cases, with low attrition, using a standardised and pre-specified method of data collection, and with little evidence of any other threats to reliability), it is possible that a study may have been excluded from our review after being rated as 0* when it could have arguably been rated as 1*. The full list of studies, including those rated as 0*, is available on the OSF repository (see Declarations for URL).

## Data Availability

The datasets generated and/or analysed during the current study are available in the OSF, https://osf.io/yu8mq/

## Appendix

See Table 7 here.

**Table 7:** Details of the studies included in the final systematic review

Authors	Publication	Participants	Context	Recruitment or retention	Intervention type	Outcome	Quality rating*
Abell et al. (2006)	Journal of Science Teacher Education	19 student teachers	USA	Recruitment	Alternative pathway/certification	Positive	1*
Allen and Sims (2017)	Education Data Lab, Wellcome Trust	18344 science teachers	UK	Retention	Training	Positive	2*
Bowe et al. (2011)	Journal of the National Association for Alternative Certification	331 student teachers	USA	Recruitment	Alternative pathway/certification	Mixed/no effect	2*
Boyd et al. (2012)	American Educational Research Journal	2693 student teachers	USA	Retention	Alternative pathway/certification	Mixed/no effect	3*
Brantlinger and Grant (2022a)	Education Policy Analysis Archives	617 mathematics teachers	USA	Retention	Alternative pathway/certification	Mixed/no effect	3*
Brantlinger and Grant (2022b)	Educational Policy	620 mathematics teachers	USA	Retention	Alternative pathway/certification	Negative	3*
Bueno and Sass (2018)	Andrew Young School of Policy Studies Research Paper Series	31171 science and maths teachers	USA	Both	Financial	Mixed/no effect	3*
Clotfelter et al. (2008)	Journal of Public Economics	103 intervention schools, 56 control schools, 29,584 teachers	USA	Retention	Financial	Positive	3*
Cooley (2009)	Proceedings of the tenth International Conference Models in Developing Mathematics Education	269 student teachers	USA	Retention	Alternative pathway/certification	Negative	2*
Demir et al. (2019)	Journal of Research in STEM Education	63 student teachers	USA	Recruitment	Financial and alternative pathway/certification	Positive	1*
Feng and Sass (2018)	Journal of Policy Analysis and Management	3294421 teachers	USA	Retention	Financial	Positive	3*
Galosy and Gillespie (2013)	A Journal of Educational Strategies, Issues and Ideas	112 teacher and 50 student teachers	USA	Retention	Alternative pathway/certification	Positive	2*
Glazerman and Seifullah (2012)	Final Report. Mathematica Policy Research	617 teachers	USA	Retention	Financial and training	Negative	3*
Kirchhoff and Lawrenz (2011)	Journal of Teacher Education	38 student teachers	USA	Retention	Training	Negative	2*
Klassen et al. (2023)	European Journal of Teacher Education	111 university students	UK	Recruitment	Training	Mixed/no effect	2*
Marder et al. (2022)	Education Policy Analysis Archives	13039 teachers	USA	Both	Financial	Mixed/no effect	2*
Morrell and Salomone (2017)	Journal of College Science Teaching	16 student teachers	USA	Recruitment	Financial	Positive	1*
Scott et al. (2006)	Journal of Science Teacher Education	37 student teachers	USA	Recruitment	Financial and alternative pathway/certification	Positive	1*
Sims and Benhenda (2022)	UCL Centre for Education Policy and Equalising opportunities	68969 teachers	USA	Retention	Financial	Positive	2*
Templeton et al. (2023)	Education Research Centre, College of Education	948 teachers	USA	Both	Financial	Negative	2*
Thompson-Lee et al. (2024)	Teaching and Teacher Education	432 university students	UK	Recruitment	Training	Positive	2*
Tomanek and Cummings (2000)	Science education	16 teachers and 15 Science majors	USA	Recruitment	Training	Mixed/no effect	1*
Urdegar (2019)	Evaluation Matters	170 teachers	USA	Both	Financial and alternative pathway/certification	Positive	1*
Whitfield et al. (2021)	Teaching and Teacher Education	29 teachers	USA	Recruitment	Financial	Negative	1*
Zahner et al. (2019)	Teachers College Record	110 teachers	USA	Both	Financial	Mixed/no effect	1*

*based on “The sieve” criteria for research quality (Gorard, 2018).

## Abbreviations

CPD: Continuing professional development
DfE: Department for education (UK government)
FIT-choice: Factors influencing teaching choice
HESA: Higher education statistics agency
KSTF: Knowles science teaching foundation
MASS: Math and science scholars program (sic)
NEU: National education union (UK)
NSLN: National STEM learning network
NYCTF: New York City teaching fellows
OSF: Open science framework
PV: Person–vocation (fit theory)
PRISMA: Preferred reporting items for systematic reviews and meta-analyses
PTTCP: Pathways to teaching careers program
RJP: Realistic job preview
SMAR2T: Science and mathematics academy for the recruitment and retention of teachers
STEM: Science, technology, engineering and mathematics
TAP: Teacher advancement program
TFA: Teach for America
TSM: Teach science and math programme
USD: American dollars
WEIRD: Western, educated, industrialized, rich and democratic
